# Small RNA Profiling of Aster Yellows Phytoplasma-Infected *Catharanthus roseus* Plants Showing Different Symptoms

**DOI:** 10.3390/genes14051114

**Published:** 2023-05-19

**Authors:** Nicoletta Contaldo, Yuri Zambon, Zsuszanna Nagyne Galbacs, Fabio Miloro, Zoltan Havelda, Assunta Bertaccini, Eva Varallyay

**Affiliations:** 1 Department of Agricultural and Food Sciences, Alma Mater Studiorum, University of Bologna, 40126 Bologna, Italy; yuri.zambon@gmail.com (Y.Z.); assunta.bertaccini@unibo.it (A.B.); 2 Institute for Sustainable Plant Protection (IPSP), National Research Council of Italy (CNR), 70126 Bari, Italy; 3 Genomics Research Group, Department of Plant Pathology, Institute of Plant Protection, Hungarian University of Agriculture and Life Sciences, Szent-Gyorgyi Albert Street 4, 2100 Godollo, Hungary; nagyne.galbacs.zsuzsanna@uni-mate.hu; 4 Plant Developmental Biology Group, Department of Plant Biotechnology, Institute of Genetics and Biotechnology, Hungarian University of Agriculture and Life Sciences, Szent-Gyorgyi Albert Street 4, 2100 Godollo, Hungary; miloro.fabio@phd.uni-mate.hu (F.M.); havelda.zoltan@uni-mate.hu (Z.H.)

**Keywords:** phytoplasma disease, small RNA HTS, miRNA profile, siRNA profile, RNA-omics

## Abstract

Micropropagated *Catharantus roseus* plants infected with ‘*Candidatus* Phytoplasma asteris’ showed virescence symptoms, witches’ broom symptoms, or became asymptomatic after their planting in pots. Nine plants were grouped into three categories according to these symptoms, which were then employed for investigation. The phytoplasma concentration, as determined by qPCR, correlated well with the severity of symptoms. To reveal the changes in the small RNA profiles in these plants, small RNA high-throughput sequencing (HTS) was carried out. The bioinformatics comparison of the micro (mi) RNA and small interfering (si) RNA profiles of the symptomatic and asymptomatic plants showed changes, which could be correlated to some of the observed symptoms. These results complement previous studies on phytoplasmas and serve as a starting point for small RNA-omic studies in phytoplasma research.

## 1. Introduction

Phytoplasmas are wall-less insect-transmitted bacteria. They strongly rely on the host during their entire lifetime and are not maintained as pure cultures in vitro, being provisionally classified in the ‘*Candidatus* Phytoplasma’ species [1,2]. Furthermore, they inhabit plant phloem and insect hemolymph. They were detected in hundreds of agronomically important plant species worldwide, which were associated with alterations in plant morphology and physiology due to modulating transcript and protein profiles, as well as changing hormone balances [3]. Phytoplasmas appear to have limited coding capacity in their genomes [4]. Moreover, they also appear to interfere with plant hormone metabolism, inducing witches’ broom, virescence, phyllody, irregular flowering, decreased fruit size, plant yellowing, decline, and stunting [2]. Developmental processes and hormone metabolism are regulated by transcriptional factors, which can be further regulated by endogenous micro (mi) RNAs, and which are associated with RNA silencing [5]. The latter is also involved in the defense mechanisms of plants, which are directed by small interfering RNAs (siRNAs). RNA silencing is a conserved mechanism of innate immunity against viruses, fungi, oomycetes, and bacteria [6]. This basal defense mechanism can control the pathogen concentration in a sequence-specific manner. Increasing the number of pathogens has been reported to encode factors that suppress RNA silencing-based host defense mechanisms [7]. Furthermore, a recent study investigated the role of a candidate phytoplasma RNA silencing suppressor (SWP16) in wheat blue dwarf disease-associated phytoplasma (‘*Candidatus* Phytoplasma tritici’) [8].

Explaining the metabolic changes allowing the symptom development during phytoplasma infection is a very important goal. In recent years, high-throughput omics has been used to study changes in phytoplasma-infected plants; gene expression changes during phytoplasma infection were characterized in pear decline-infected pear [9], metabolome changes were described in sweet cherry showing virescence [10], and biochemical and transcriptomics were used to study the differences between aster yellows-infected cucumber and healthy cucumber [11]. A comprehensive high-throughput RNA-Seq and sRNA-Seq study of grapevine infected with ‘*Ca.* P. solani’ revealed different changes in the plant response to phytoplasma presence in different growing seasons [12], while dynamic DNA methylation patterns were characterized in phytoplasma-infected mulberry [13] and paulownia plants [14]. Significant changes in the miRNA profile were demonstrated during the phytoplasma infection of different plant hosts, such as Mexican lime, mulberry, paulownia, Chinese jujube, and grapevine [15,16,17,18,19,20,21].

*Catharanthus roseus*, the model plant for phytoplasma research, is also a medicinal plant that produces large quantities of monoterpenoid indole alkaloids, which are used in chemotherapy. In addition, vinblastine and vincristine are beneficial for humans and are the end products of the terpenoid indole alkaloid (TIA) pathway. TIA expression can be finely tuned by several genes induced by ORCA2, ORCA3 [22,23], and DAT/MAT/D4H-like genes [24]; meanwhile, TIA expression is repressed by the ORCA3-induced expression of CR1 [25]. During pathogen infection, the ORCA3 level was reported to be induced, thus resulting in an altered TIA pathway. Several genes involved in these pathways are specifically expressed in a few tissues. Although the miRNA profiling of *C. roseus* is available [26,27,28], there are no studies reporting the changes in the small RNA expression patterns during phytoplasma infection.

In this work, the small RNA expression profile of plant clones from originally phytoplasma-infected plants showing different symptoms is reported. Besides the comparison of the symptoms and their correlation with the phytoplasma concentration, small RNA expression patterns related to the presence of a ‘*Ca*. P. asteris’ strain (aster yellows, AY) in *C. roseus* plants showing virescence (Vir) or witches’ broom (WB) symptoms in comparison with those of asymptomatic (NO) plants are studied.

## 2. Materials and Methods

### 2.1. Plant Material

**Figure 1:**
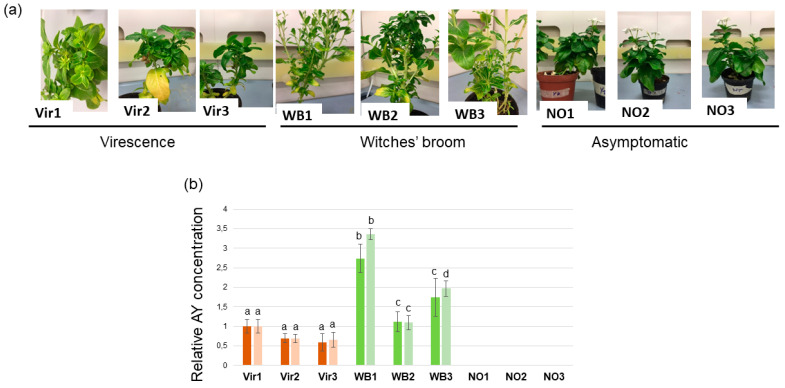
(**a**) Photos of the selected periwinkle plants showing virescence (Vir) symptoms, witches’ broom (WB) symptoms, and no symptoms (NO). (**b**) The relative AY DNA concentration was determined via qPCR analysis in the different plants. (**b**) The phytoplasma quantification was performed on three DNA extracts for each plant via qPCR assay. Graphs show the relative quantification of the DnaA (stronger color) and the DnaB (lighter color); these were normalized on both CrUBQ11 and CrEF1α via LightCycler^®^ 96 Software (Roche, Basel, Switzerland). Data are presented as the mean ± SD (n = 3). Statistical analysis was performed, via one-way ANOVA, only between the plants showing an infection phenotype. Multiple comparison tests were conducted using Tukey’s test and were marked using a compact letter display (CLD). The samples were labeled with the same letter and were not significantly different from each other.

*C. roseus* plants were infected with ‘*Ca*. P. asteris’ (strain Hyd35, 16SrI-B) [29,30]. Cuttings from the original plant were micro-propagated, kept in vitro for several years, and potted in April 2016. After about 6 months, they showed three phenotypes, including virescence symptoms, witches’ broom symptoms, and no symptoms (Figure 1). Three plants from each group were chosen for further analyses and grown in the same glasshouse, specifically under insect-proof conditions at 23 °C and 12 h light/dark cycles per day.

### 2.2. Phytoplasma Detection and Quantification

In order to verify the presence and identity of phytoplasmas in the selected plants, total nucleic acid extraction was carried out following the method of White and Kaper [31]. Total nucleic acid extracts were used for the small RNA HTS described below. In addition, these were employed for phytoplasma quantitative PCR analyses for the purposes of detecting two genes of AY, the DnaA gene (chromosomal replication initiator protein DnaA—AYWB_RS00005) and the DnaB gene (replicative DNA helicase DnaB—AYWB_RS00035). The analysis was conducted in triplicate for both genes using the periwinkle elongation factor 1-α (CrEF-1-α) and ubiquitin (CrUBQ11) as the internal control (Table S6). The quantitative PCR was performed in a LightCycler 96 instrument (Roche, Basel, Switzerland). The amplification reactions were carried out using a Luminaris Color HiGreen qPCR Master Mix (ThermoFisher, Waltham, MA, USA) and 50 ng DNA as the template. A 2-step amplification protocol, as suggested by the manufacturer, was used. The results were then analyzed via the 2^∆∆Ct^ method (specifically using the embedded software of the LightCycler 96 instrument (Roche, Basel, Switzerland)), using both CrEF-1-α and CrUBQ11 as the reference genes.

### 2.3. Small RNA HTS

A small RNA fraction was purified from the extracted total nucleic acid and used for a small RNA sequencing library preparation. In total, nine libraries were prepared using the TruSeq Small RNA Library Preparation Kit (Illumina, San Diego, CA, USA). A modified protocol was used [32], whereby the sequencing was conducted using a single index on a HiScan2000 via UD GenoMed (Debrecen, Hungary), which was 50 bp long and with a single end reading. The FASTQ files of the sequenced libraries were deposited into the NCBI GEO database and can be accessed through the series accession number GSE213754.

### 2.4. Bioinformatic Analysis of the Small RNA Sequences

For the bioinformatics analysis, a CLC Genomic Workbench (Qiagen, CLCbio, Aarhus, Denmark) was used. After quality control, the trimming and removal of the *C. roseus* non-coding RNA (rRNAs and tRNAs) (RNAcentral) was performed. In addition, the small RNA reads were mapped to the *C. roseus* genome (ASM94934v1) [33]. The reads that were not mapped on the *C. roseus* genome were subsequently mapped on the genome of one strain of the aster yellows phytoplasma, including its published four plasmids [34]. The percentage of the mapped reads and the coverage of the AY genome by them was calculated in each library. Then, the samples were averaged out according to the phenotype. The *C. roseus*-mapped small RNAs were further analyzed for the presence of published miRNAs. For this analysis, first, the *C.*-*roseus*-annotated miRNA database [35] was used. For the annotation of the miRNAs that were not annotated there, we used miRBase (release 22.1) data for the *Arabidopsis*, tomato, and soybean miRNAs present at miRBase.

The relative expression values of the miRNAs and siRNAs were generated using the R (4.1.0) package pheatmap (1.0.12) [36]. The heatmaps were prepared using z-scores, which were calculated by RPM (reads per million). The hierarchical clustering was performed for the rows, and the gene clusters were defined by cutting the dendrograms in the groups. The principal component analysis (PCAs) of the miRNAs and siRNAs were made using the web tool ClustVis [37]. Only the miRNAs with a ≥5 mean RPM and siRNAs with a ≥50 mean in at least one group were taken into consideration for the data in the heatmaps and PCAs.

### 2.5. Small RNA Northern Blot

For the small RNA Northern blot analyses, the total RNA samples (5 µg) were fractionated on denaturing 12% polyacrylamide gels, which contained 8 M urea, and were transferred to Nytran NX membrane (GE Healthcare, Chicago, IL, USA) via a capillary method using 20xSSC, and were fixed by ultraviolet cross-linking. Membranes were probed with radioactively 32P-labeled LNA oligonucleotide probes (Exiqon, Vedbæk, Hovedstaden, Denmark), which were complementary to the mature microRNAs (Table S6), using a published protocol [38]. Briefly, 5 pmol of each oligonucleotide probe was end-labeled with [γ32P] ATP by using T4 polynucleotide kinase. The prehybridization of the filters was carried out in 50% formamide, 0.5% SDS, 5xSSPE, 5x Denhardt’s solution, and 20 µg/mL of sheared denatured salmon sperm DNA. The hybridization was carried out at 50 °C for 2 h in the same buffer. The washing of the membranes was performed for 10 min, two times, with a washing solution containing 0.1%SDS and 2xSSC at the hybridization temperature.

### 2.6. Reverse Transcription Quantitative Polymerase Chain Reaction (RT-qPCR) Analysis of the Predicted Target Genes

RT-qPCR analysis was carried out, as described in [39], in order to verify the differential expression of the selected genes that are targeted by miRNAs with seriously altered expressions in the phytoplasma-infected plants. The M-MLV reverse transcriptase (Promega, Madison, Wisconsin, USA) was employed in order to synthesize the cDNAs by using a random hexamer primer (Thermo Fisher Scientific Baltics, Lithuania) by following the manufacturer’s instructions. For all of the genes of interest, ~1.5 ng of a cDNA template was used in qPCR with the expression normalized to the ubiquitin gene. The qPCR reactions were performed using the SYBR Green Master Mix (Applied Biosystems, Foster City, CA, USA). This was achieved by using gene-specific primers (Table S6) and a RealTime PCR ABIPRISM StepOne sequence detection system (Applied Biosystem, Foster City, CA, USA) in three technical replicates and three biological replicates. The relative quantifications in gene expression were determined using the 2^ΔΔCt^ method, thereby obtaining the fold changes in the gene expression, which were normalized to CrUBQ11, a polyubiquitin gene. The mean data obtained were analyzed by ANOVA (P 0.05), followed by Student’s *t*-test.

## 3. Results

### 3.1. Phytoplasma Concentration in Periwinkle Plants

*C. roseus* were initially infected with ‘*Ca*. P. asteris’. The cuttings from this plant were maintained in micropropagation for years, then potted. In the following year, these plants showed three phenotypes, including virescence (Vir), witches’ broom (WB), and no symptoms (NO) (Figure 1). Three plants (Vir1–Vir3) showed virescence, an abnormal development of green pigmentation occurring in the flowers, which is why the originally white flowers became green. The structure of the flowers was differently maintained; the symptoms of the Vir1 plant were the most severe. The flowers of this plant not only showed virescence, like the flowers of Vir 2 and Vir3, but also showed slight phyllody, which is when the flower structure starts to become leafy. Three plants (WB1–WB3) showed the witches’ broom phenotype. Besides extensive branching, their internodes shortened, leading to a slightly stunted phenotype of the branched shoots. In addition, they did not flower, or their flowers turned green. The witches’ broom symptom was the most severe in the case of WB1 followed by WB3; although WB2 showed severe branching, its leaves stayed bigger and their stunting was negligible. Six plants of the originally infected periwinkle descendant were asymptomatic. They had no unusual branching or stunting, and their flowers stayed white, with the original flower structure maintained. All the symptomatic plants and three plants from those in the asymptomatic group were chosen for the analysis (Figure 1a and Figure S1).

To better verify the phytoplasma infection stage, the bacteria titer was determined. Aster yellows phytoplasma (AY) infections were detected in the Vir and WB plants showing symptoms and was only detected in incredibly low concentrations in one of the recovered plants (NO1) determined via qPCR assays calculating the delta–delta Ct, using the *C. roseus* elongation factor 1 and ubiquitin gene as references (Figure 1b). The phytoplasma DNA concentrations in the WB plants was higher than the concentrations in the Vir plants; however, the concentration was about the same in the Vir1 and WB2 plants. The concentration of phytoplasma DNA was very low in the NO1 plant, and no phytoplasma was detected either in the NO2 or NO3 plants.

### 3.2. Small RNA HTS Profiling

From the Vir, WB, and NO plants, nine small RNA sequencing libraries were prepared and sequenced, thereby revealing 14.8–27.7 million reads, resulting in 14.4–25.3 million redundant and 3.3–5.4 million non-redundant reads after trimming and demultiplexing (Table S1). The size distribution of the sequenced small RNAs showed a typical pattern. The majority of both the all-genome-mapped and the *C. roseus*-genome-mapped reads were 21 and 24 nt long, respectively. This demonstrates that they are products of the plant DICER enzymes, and that they also show that the RNA used for the library preparation was not degraded (Figure S2a,b).

A total of 11–20 million reads could be mapped to the *C. roseus* genome (Table S2), and the reads were analyzed for the miRNA and siRNA expression, while the remaining reads were mapped to the AY phytoplasma genome [34].

### 3.3. Characteristics of Phytoplasma-Derived Small RNAs

**Figure 2:**
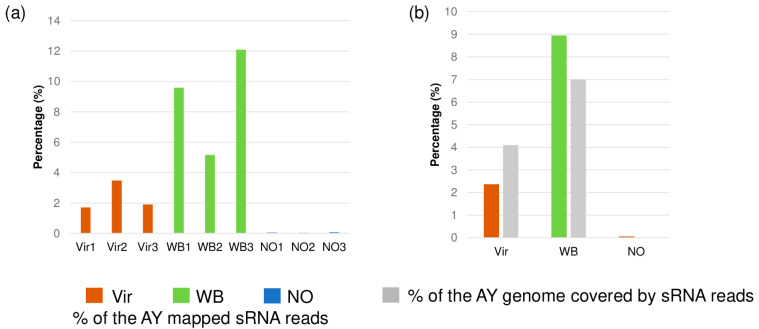
The distribution of the AY-derived reads in the phytoplasma-infected plants. In (**a**), the percentage of the small RNAs that could be mapped to the AY phytoplasma genome in each of the libraries is shown. In (**b**), the average percentage of the AY-mapped small RNAs and the coverage of the AY phytoplasma genome in them are shown.

The small RNA reads that were not mapped to the *C. roseus* host genome were mapped to the AY genome. The analysis showed that for the Vir and WB plants, 1.7–3.4% and 5.1–12% of the total small RNA reads could be mapped to the phytoplasma genome, while for the asymptomatic plants, this value was 0.03–0.07% (Figure 2a).

Most of the small RNAs could be mapped to both of the two 16S rRNA gene copies that are encoded in the AY genome, but they were also mapped to different parts of the phytoplasma genome. The coverage of the AY genome by the small RNAs was 4.1% and 7% in the Vir and WB plants, respectively (Figure 2b). There was only a trace amount of AY-derived small RNAs in the NO plants, which were mapped to the conservative part of the 16S rRNA encoding gene. The WB plants had both a higher number of AY-mapped reads and a bigger coverage of the AY genome when compared to the Vir plants. To investigate whether these AY-derived small RNAs could have a specific size property reflecting their origin, the size distribution of the AY-mapped reads was checked (Figure S3). The size distribution of the AY-derived reads showed a constantly decreasing length.

### 3.4. The miRNA Profile of the Plants, Showing Different Symptoms

**Figure 3:**
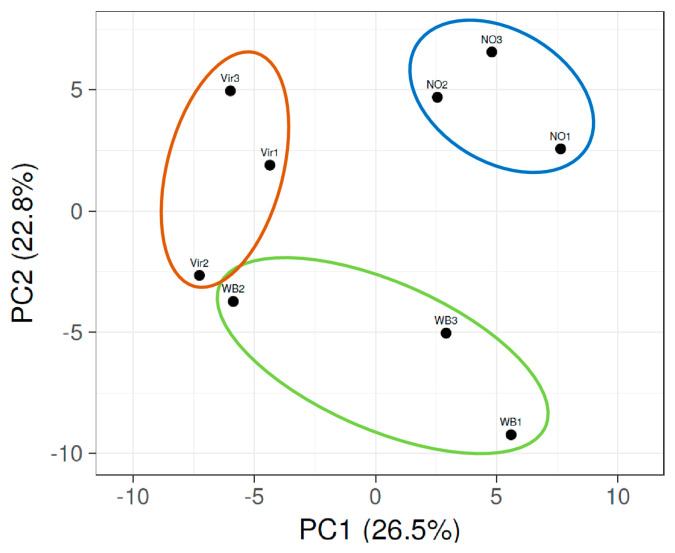
The principal component analysis of the expression changes in the miRNA profile of the nine small RNA libraries, as prepared from the plants showing different symptoms (Vir, WB, and NO). Color circles highlights PCAs of the three groups of plants.

The miRNA profiling of the sequenced reads was performed on the reads that could be mapped to the *C. roseus* genome. The reads per million (RPM) were calculated for all the individual small RNA sequences. In order to be able to focus on the changes that could play a role in the symptom development, those having a high expression (at least 50 counts per million reads) and a significant expression change (higher than two times) were selected (Table S2). The principal component analysis (PCA) of the miRNA profiles showed that the NO plants clustered together, while those of the plants infected with phytoplasmas and showing different symptoms had a distant correlation from them (Figure 3).

**Figure 4:**
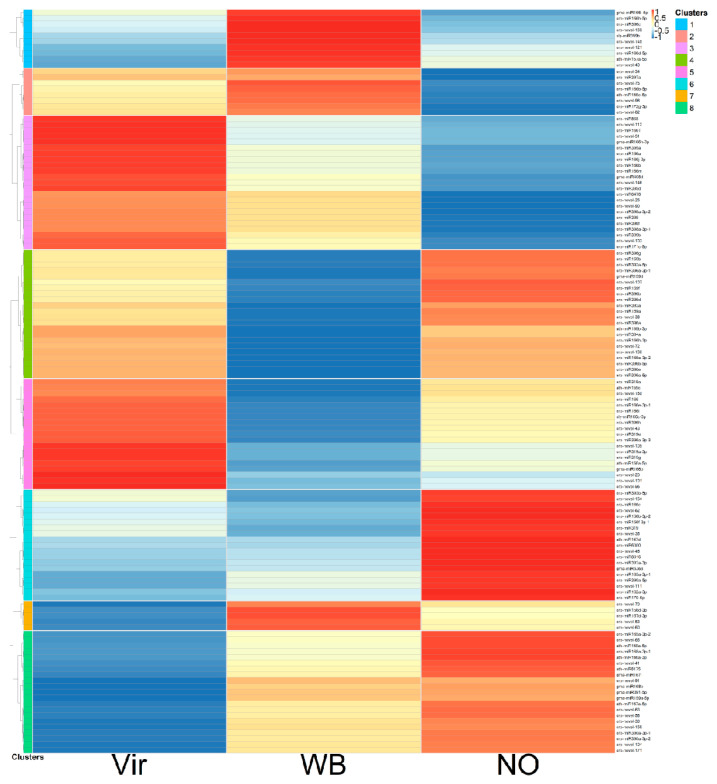
A heatmap of the average expression changes in the conserved and novel miRNAs in the different plant groups according to their z-scores. Detailed list of the miRNAs presented in the figure, together with their z-scores, can be found in Table S3.

The Vir2 and WB2 plants had about the same percentage of the AY reads and were the closest in this analysis. Specific changes in the miRNA expression levels were visualized by preparing a heatmap of the z-scores (Figure 4).

The z-score changes were markedly different between the three groups of plants and could be grouped into eight clusters. Some of the clusters showed similar trends (clusters 4 and 5, and 6 and 8). It was interesting to note that although there were clusters showing the same tendency in the Vir and WB groups when compared to the NO samples (clusters 2, 3, and 6), there were also clusters in which the miRNA changes were more different between the Vir and WB groups than between the Vir and NO groups, or between the WB and NO groups (clusters 1, 4, 5, and 7). Detailed data for the aforementioned are presented in Table S3.

The z-scores’ heatmap shows a differential regulation, but this representation does not take into consideration the absolute expression level of the individual miRNAs. To highlight the changes in relation to the expression levels, a new list was prepared grouping the samples according to the miRNA families (Table S4). In several cases, the different forms of the same miRNA family clustered into a different group. The summarized data of the most important changes, which showed the expressions of the most abundant members of the miRNA family, are shown in Table 1.

In order to validate the small RNA HTS analysis, the expression of some of the conservative miRNAs was investigated by a Northern blot hybridization, using LNA probes. For this validation, the RNA samples from individual plants were used.

The expression of miR156 showed a slight increase in the Vir and a slight decrease in the WB plants, but different miR156 species exhibited a slightly different pattern. The validation of its expression in the leaves shows a slight decrease in the Vir2 plants, but an increase in the WB2 plants, when compared to the NO2 plants. The Vir2 and WB2 groups were the less typical plants in their group, and this could explain the difference between the summarized small RNA HTS and the hybridization results (Figure S4a,b). Two types of mir157 were identified, and only the less abundant one had an altered expression, showing a down-regulation in the Vir plants and an up-regulation in the WB plants. The Northern blot validation, while using RNA that was extracted from the stems, confirmed this altered expression (Figure S4c). Several different types of miR159 were identified by the HTS with slightly altered expression patterns and rates, showing an overall decreased expression pattern in both the Vir and WB plants. The Northern blot analysis showed a slightly different pattern, and it also showed an increased expression in the phytoplasma-infected plants (Figure S5). The mir162 expression was down-regulated in both the Vir and WB plants. The expression of the two types of miR165 and the several miR166 family members was altered. Although several miR166 members showed an increased expression rate, these changes were masked by the highly expressed miR166 species (showing reads that were higher than 100,000 RPM when compared to 5–50) (Table 1 and Table S5). The Northern blot analysis was conducted for the miR166, and its decreased expression rate was validated (Figure S6). The miR167 expression showed a significant down-regulation in the phytoplasma-infected plants, which was also confirmed by the Northern blot analysis (Figure S5c). The expression of miR168 showed a decrease in the phytoplasma-infected plants. This decrease was more severe in the Vir plants and was validated by the Northern blot hybridization (Figure S7). The miR168 was annotated incorrectly on the CroFGD website, whereby the reported mature form was actually the star strand. The mature form of the miR168 was found to be identical to that of *Arabidopsis thaliana*; however, when using this correct sequence of miR168, no target was found in the CroFDG’s CDS collection. The miR168 regulates ARGONAUTE 1 (AGO1) mRNA, but the AGO1 sequence, which is annotated in the CroFGD (accession number CRO_T016560), was only partial. When using the more recent genome of *C. roseus* (ASM2450571v1), it was possible to identify the entire AGO1 gene sequence and to obtain more accurate predictions of the CDS and protein sequences. A new miRNA target prediction was performed using psRNATarget [40] with miR168 and CrAGO1, whereby the analyses confirmed the possibility of their interaction. The result indicated that the target region for the miR168 was identical to the one in *A. thaliana*, thus confirming the miRNA–target interaction in the CrAGO1 (Figure S7c).

The expression of the miR170 was decreased (Table S4), while the expression of the miR171 was increased in the phytoplasma-infected plants, which was validated by the Northern blot analysis (in this latter case of WB2 (Figure S8)). The miR172 expression showed an increase in both the Vir and WB plants, but this change could not be fully validated either in the Vir or in the WB plants from the RNA that was purified from the stems (Figure S9). The miR319 showed a slightly increased expression in the Vir plants, and a slight decrease in the WB plants, but only this later alteration could be validated (Figure S5d). The miR390 and miR391 both showed a decreased expression in the phytoplasma-infected plants. Moreover, this was validated in the case of miR390 by the Northern blot analysis (Figure S10). Although the signal was very faint, this result was in line with the low RPM; in addition, it was visible that, in the phytoplasma-infected plants, this signal was decreased.

Although the miR395 was identified with a low RPM, its expression changed very drastically (mostly in the Vir plants). The expression of the miR396 decreased due to phytoplasma infection, and this decrease was stronger in the WB plants (Table S4). Although a pronounced signal for this RNA was expected, only a faint signal during the Northern blot validation was obtained, due to not being able to unambiguously confirm its decreased level (Figure S11). The miR398 showed a strongly increased expression in the phytoplasma-infected plants, which was validated by the Northern blot analysis (Figure S12).

The expression changes in several currently described *C. roseus*-specific miRNAs [26] were found in the phytoplasma-infected plants. Among them, cro-novel-34 was highly expressed in the asymptomatic plants (about 1000 RPM) and was significantly induced (more than twice) in both the Vir and WB plants.

### 3.5. The siRNA Profiles of the Investigated Plants

**Figure 5:**
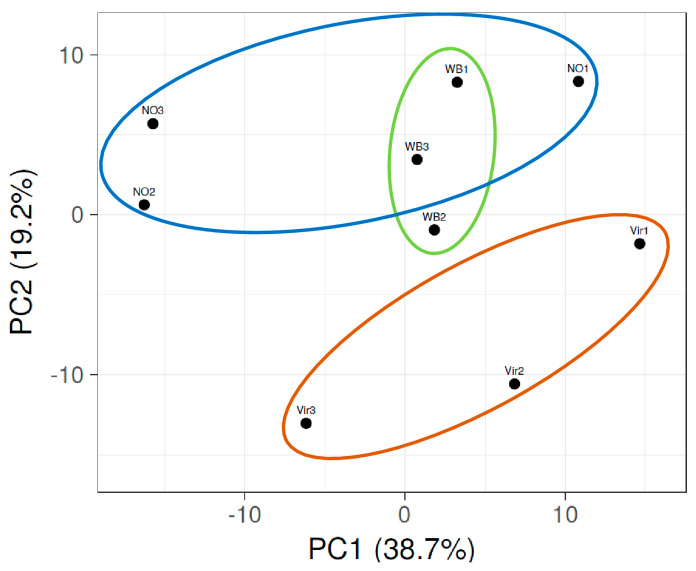
The principal component analysis of the expression changes in the siRNA profile in plants showing virescence (Vir) symptoms, witches’ broom (WB) symptoms, or no symptoms (NO). Color circles highlights PCAs of the three groups of plants.

Small RNAs, which were involved in the siRNA pathway, were also analyzed to identify whether phytoplasma infection could change their expression. The 24 nt long reads mapped to *C. roseus* were selected. To focus on the relevant changes, only the siRNAs presented with at least 50 RPM in one of the three groups of plants were analyzed. As an outcome, a total of 305 unique 24 nt long siRNAs were identified. Among these sequences, 22 belong to the ribosomal RNAs or transfer RNAs. The other siRNAs exhibited predominantly 5′adenine (about 66%), which is in accordance with the preferential recruitment of the AGO4 and AGO6 in *Arabidopsis* [41], i.e., the key proteins involved in the DNA methylation pathway. The PCA analysis showed that both the Vir and WB plants cluster together, where the WB plants were particularly closely related (Figure 5).

**Figure 6:**
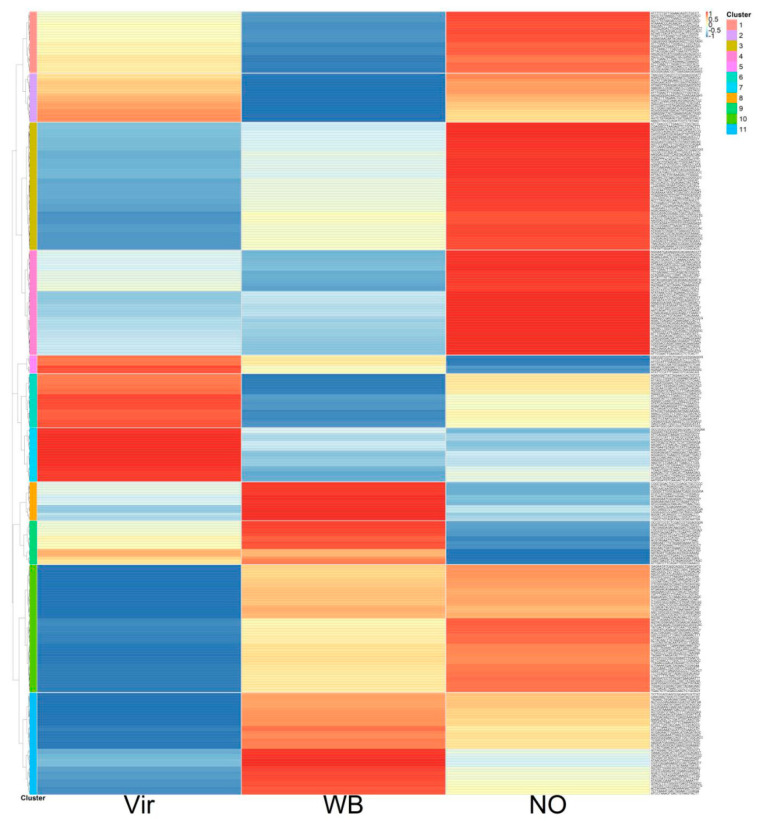
A heatmap of the average expression changes in siRNAs in the different plant groups according to their z-scores. Detailed list of the miRNAs presented in the figure, together with their z-scores, can be found in Table S5.

NO1, the asymptomatic plant in which phytoplasma DNA was detected, clusters closer to the WB group than to the other two asymptomatic plants. The detailed analysis and heatmap obtained from the average RPM of the three plant groups were converted to z-scores. This showed that the siRNA clustered into 11 groups, with different expression patterns (Figure 6).

Among those phytoplasma-responsive siRNAs, 53 were significantly decreased (fold change ≥ 2), and only 1 was up-regulated in the Vir plants. In the WB plants, 16 siRNAs were significantly decreased, and 10 siRNAs were up-regulated (Table S5).

**Figure 7:**
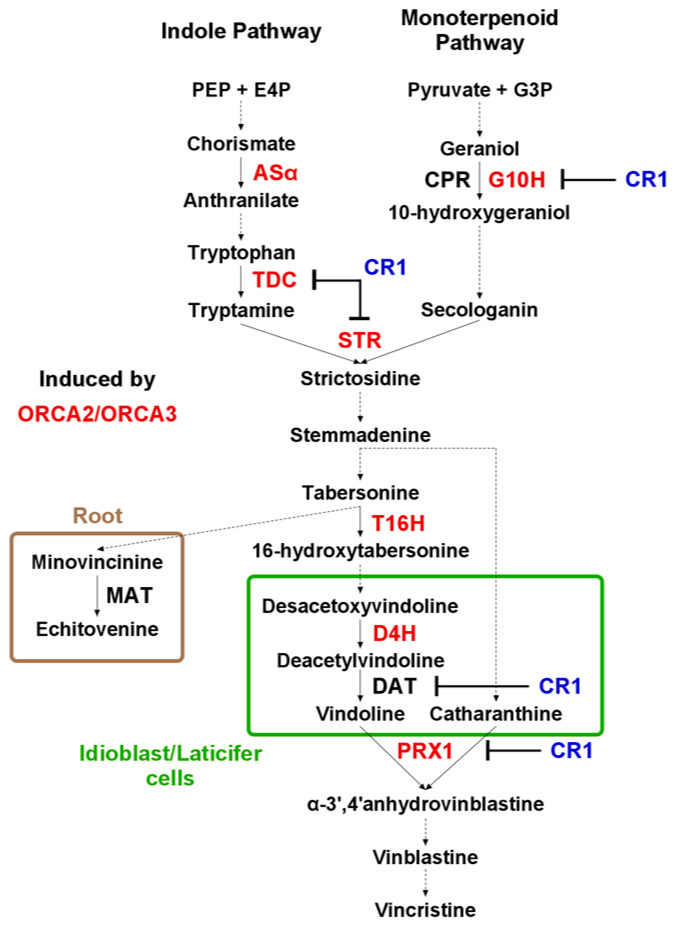
A schematic representation of the TIA pathway. Enzyme abbreviations are written in capital letters next to the arrow, indicating the reaction that was catalyzed by each enzyme. Red colored enzymes are up-regulated by either, or both, ORCA2 and ORCA3. The inhibition of the enzyme by CR1 are directly indicated. The metabolites resulting from different enzymatic conversions are indicated by the appropriate arrows. The solid arrows represent single enzymatic conversions, whereas dashed arrows indicate multiple-step or uncharacterized enzymatic conversions. Asα (anthranilate synthase); TDC (tryptophan decarboxylase); CPR (cytochrome P450 reductase); G10H (geraniol 10-hydroxylase); STR (strictosidine synthase); T16H (tabersonine 16-hydroxylase); MAT (minovincinine-19-*O*-acetyltransferase); D4H (desacetoxyvindoline 4-hydroxylase); DAT (deacetylvindoline 4-*O*-acetyltransferase); and PRX1 (vacuolar class III peroxidase).

During the siRNA analysis, several detected siRNAs were mapped to the upstream region of the minovincinine 19-hydroxy-*O*-acetyltransferase (CrMAT—GenBank accession number AF253415) [24] and desacetoxyvindoline-4-hydroxylase-like (CrD4H-like—GenBank accession number GU363550) [42] genes (Figure 7).

**Figure 8:**
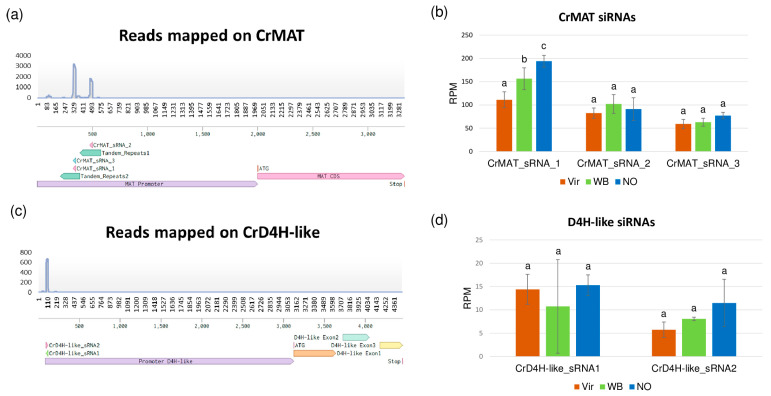
A schematic representation of the siRNAs that were mapped to the promoter region of the (**a**) CrMAT and (**c**) CrD4H-like genes. Regarding the left side, the expression rate of the siRNAs from the Vir, WB, and NO plants are shown as a column diagram (**b**) for CrMAT (**d**) for CrD4H-like genes. Two-way analysis of variance (ANOVA) and pair-wise Tukey HSD test results for the different quantity of siRNAs on the promoter of CrMAT and CrD4H-like genes. The samples labeled with the same letter were not significantly different from each other.

CrMAT and CrD4H-like are involved in the alternative pathway of vindoline synthesis, where their altered expression could affect the synthesis rate of vindoline, which is one of the main *C. roseus* products used in medicine. CrMAT has been reported to be active only in the root; thus, siRNAs targeting its promoter are possibly able to alter its expression and could explain its reduced expression in the leaves. In the CrMAT gene, siRNAs are mapped to the predicted promoter region that contains tandem repeats. The expression of the most abundant siRNAs decreased with 30% and 11% in the Vir and WB plants, respectively. The siRNAs mapped to a D4H-like gene also show similar behavior, although their combined expression is only slightly higher than 25 RPM in the control. The two main siRNAs are derived from the same sequence element, but with opposite orientation, showing their real siRNA origin (Figure 8).

### 3.6. Investigation of the TIA Pathway Key Regulator Expression during Phytoplasma Infection

**Figure 9:**
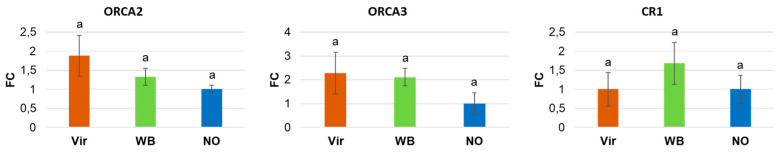
The gene expression analysis of the ORCA2, ORCA3, and CR1 enzymes playing a role in the terpenoid biosynthetic pathway via RT-qPCR. The column diagram shows the relative expression determined via qRT-PCR and ubiquitin as an endogenous control via LightCycler^®^ 96 Software (Roche, Basel, Switzerland). Data are presented as the mean ± SD (n = 3). Statistical analysis was performed, via one-way ANOVA, only between the plants showing an infection phenotype. Multiple comparison tests were conducted using Tukey’s test and are marked using a compact letter display (CLD). The samples labeled with the same letter were not significantly different from each other.

Although, due to the lack of a proper annotation of the *C. roseus* genome, a prediction analysis of the possible miRNA target genes was not carried out, the expression pattern of certain key enzymes playing a role in the terpenoid biosynthetic pathway was investigated. ORCA2, ORCA3, and CR1 are key regulators of the TIA pathway. Meanwhile, the ORCA2 and ORCA3 promote the CR1 blocks, the transcription of terpenoid indole alkaloid (TIA) genes encoding molecules that are produced in plants under stress (Figure 7) [43]. They can be regulated by jasmonate-responsive AP2-domain transcription factors, which are regulated by the miR172. The mir172 expression was induced in the Vir and WB plants. Although there is no evidence of a direct correlation between the miR172 and the above three genes, we thought that it would be important to know how their expression was changed during phytoplasma infection. The expression of ORCA2 and ORCA3 were slightly induced in the Vir and WB plants, while the expression of CR1 was only induced in the WB plants (Figure 9).

**Figure 10:**
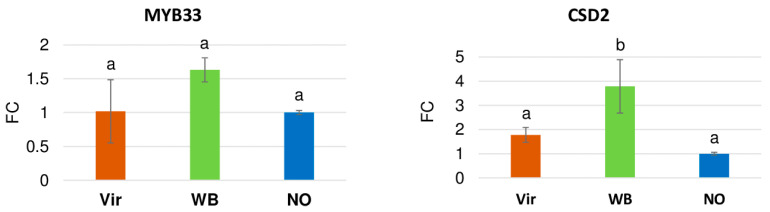
The expression analysis of MYB33 and CDS2 via RT-qPCR. The column diagram shows the relative expression determined via qRT-PCR and with ubiquitin as an endogen control, with the use of LightCycler^®^ 96 Software (Roche, Basel, Switzerland). Data are presented as the mean ± SD (n = 3). Statistical analysis was performed, via one-way ANOVA, only between the plants showing an infection phenotype. Multiple comparison tests were conducted using Tukey’s test and are marked using a compact letter display (CLD). The samples labeled with the same letter were not significantly different from each other.

MYB33, as a member of the MYB superfamily, plays a role in plant hormone responses during the defense against pathogens [44]. In addition, it exists under the miR159 regulation, and its expression was slightly induced in the WB plants (Figure 10). The same trend of changes occurred, where the highest expression in the WB plants was observed for the superoxide dismutase, CSD2, which is the key enzyme in ROS detoxification and is regulated by the miR398 [45] (see Figure 10).

## 4. Discussion

In this work, the small RNA profile of nine originally phytoplasma-infected *C. roseus* plants was studied. While six plants showed phytoplasma symptoms of virescence and witches’ broom, three plants were asymptomatic. The phytoplasma symptoms can show remission, especially after long-term micropropagation; this status is usually temporary, and symptoms usually re-emerge (A. Bertaccini, unpublished data). One of the asymptomatic plants was PCR positive but had significantly lower phytoplasma concentration when compared to the symptomatic plants. Quantitative PCR showed significantly high phytoplasma concentrations in the symptomatic plants, which was typically higher in the WB plants than in the Vir plants. The phytoplasma concentration in the Vir1 plant, which was the most symptomatic plant in the Vir group, and the WB2 plant, i.e., the least symptomatic plant in the WB group, were about the same, thus suggesting that the phytoplasma concentration in the leaves correlates with the symptom intensity, which is in agreement with previous reports [46]. For the study, instead of uninfected plants, the recovered plants were chosen as a control because they originated from the same plants, so their genetic expression was identical. The miRNA pattern of the plants can be altered by slight changes in the environment, and the aim was also to minimize the uncontrolled changes. The recovered plants that were the descendants of the same plants and underwent the same propagation, and were maintained at the same place, consequently differed from the symptomatic plants only in the presence of the symptoms, which turned out to be correlated very well with the AY concentration. In this case, the difference in the miRNA and siRNA profile compared to the symptomatic plants could be undoubtably coupled to the higher concentration of the phytoplasma. The phytoplasma-derived small RNAs were present in the phytoplasma-infected plants, raising the possibility of their use in pathogen diagnostics. However, as their size distribution did not show an abundance of 21–24 nt long small RNAs, they are very likely not products of specific DICER enzyme activity, but are more likely products of other RNase enzymes, which are active in the *C. roseus* host.

The PCA analysis shows that the miRNA pattern in the phytoplasma-infected symptomatic plants was different from those of the asymptomatic plants. Consequently, these different expression patterns can also play a role in the different observed symptoms.

The z-score display of the miRNA expressions showed eight distinguishable clusters. Their analysis suggests that there are differentially regulated miRNAs comparing not only the NO and phytoplasma-infected groups, but also comparing the WB and Vir groups, since their different expression is present in plants with different symptoms.

The changes in the miRNA expression were detected by the small RNA HTS, which were further investigated via the Northern blot analysis in some cases. While the small RNA HTS results were analyzed by the mean of the three different groups of plants showing different phytoplasma concentrations and symptoms, the Northern blot analysis was carried out with the use of the RNA extracts of individual plants via LNA probes that were used to detect the conserved members of the various miRNA families. The two methods showed the same trend in most but not in all cases. There are only a limited number of reports investigating the changes in the miRNA profile of the phytoplasma-infected plants. In these reports, a small RNA profiling of Mexican lime, which was infected with ‘*Ca*. P. aurantifolia’; mulberry, which was infected by aster yellows phytoplasmas; paulownia, which was infected with paulownia witches’ broom; and a witches’ broom-diseased Chinese jujube was carried out and based on the data from only the one–one libraries, which were prepared from the infected and healthy plants [15,16,17,18,19]. Although in the infected libraries, the samples from the different individuals were pooled, these limited sources of data could explain the differences in their results. Additionally, small RNA profiling was conducted on the grapevine var. Chardonnay, which was infected by either aster yellows phytoplasma [20] or ‘flavescence dorée’ [21]. The different observations of these two works suggest that the effects of different phytoplasma strains in the same host species can be different. As the pathogen presence is limited to the phloem, its effect could be also different in the stems and in the distinct parts of the plant [47]. Keeping in mind the limits of these techniques, certain conclusions can be drawn from the results of the work presented here.

Several studies in annual and perennial plants have identified the conserved roles of miR156 and its targets, i.e., SBP/SPL genes, in guiding the switch of plant growth from juvenile to adult phases. The miR157 is thought, as is the case in the miR156 family, to target mRNA coding proteins containing the Squamosa promoter binding protein (SBP) box [48]. However, neither the miR157 nor the miR156 have targets that are predicted in *C. roseus*; instead, they are thought to target ten mRNAs in other plant species that are fine-tuning the plant developmental processes. Their altered expression in the phytoplasma-infected plants suggests that they could play a role in the specific symptom development, as it was found in most of the cases when miRNA profiling was investigated in phytoplasma-infected plants [15,16,17,18,19,21].

The mir159 and miR319 share a common target, a MYB transcription factor, which is annotated in *C. roseus*. Moreover, the miR319 targets are members of the TCP gene family, which participate extensively in plant development and in responses to environmental stresses. In sepals, petals, and anthers, it was found that the GAMYB and TCP proteins were expressed and that they directly interacted with regulating miR167, which creates a miR159–miR319–miR167 network [49]. The expression of the miRNAs in this network was found to change during phytoplasma infection, but its induction and repression were also both reported. In this network, the concentration of the miR167 was kept low, thereby resulting in a strong ARF6/8 expression, which, in turn, regulates many of the genes that are required for flower development, including auxin signaling regulation. In the experiments performed here, both the miR159 and miR319 were down-regulated in the phytoplasma-infected plants, thus leading to a down-regulation of the miR167. This results in a disturbance in the ARF-regulated flower development, which could result in the virescence phenotype. The expression of the MYB33 gene was shown to be increased in the WB plants, whereas the expression of the miR159 was down-regulated, thereby suggesting a direct miRNA–target correlation.

The mir165/166 family represents the most abundant miRNAs in plants, having nine members in *A. thaliana* and in their targeting of five members of the HD-ZIP III transcription factor family, as well as in controlling developmental processes, such as apical shoot development, through the expression of several auxin biosynthesis, transports, and response genes [50]. As they show differential patterns in the phytoplasma-infected plants, it is highly possible that their altered expression can shape the developmental changes that lead to specific symptom development. In the data presented here, in contrast to the other species [15,17,18], the expression of all the miR166 species, in total, was down-regulated. However, it is possible that this overall expression change was masked because of the high number of the reads that can be different in distinct tissues of the plant, thus resulting in an altered regulation mechanism.

In its canonical form, the miR162 targets DCL1; meanwhile, the miR168 targets AGO1, which are the main executor molecules of the miRNA pathway. For the miR162, no target could be predicted in the *C. roseus*, but its over-expression or blocking its effect on the target induced resistance in *Oryza sativa*. In addition, in its pathogen-susceptible variants, a decrease in the miR162 could be detected [51]. Its level was decreased in both the Vir and WB plants, which suggests an efficient pathogen counter-defense strategy, but this phenomenon should be further investigated for better clarity. The AGO1 level was finely tuned by a precise miR168 concentration, which also showed an altered expression during phytoplasma infection. In previous works, it was found that during virus infection, the increased miR168 concentration can translationally inhibit the AGO1 expression and can also decrease the activity of antiviral silencing [52]. During phytoplasma infection, the concentration of miR168 decreases, which can result in an increased AGO1 level and activity; however, this hypothesis should be further addressed.

The miR170 and mir171 target SCARECROW-like genes, which are a family of transcription factors that play a role in gibberellin-controlled radial patterning in roots. Their altered pattern in the phytoplasma-infected plants were detected and, in the case of the miR171, it was validated by the Northern blot analyses. The increased expression of the miR171 could be detected in the WB2 plants; meanwhile, in the Vir2, the less typical plant showing virescence, this increase could not be seen. The increased expression of the miR171 was reported previously from *Ziziphus jujube* and mulberry [16,19], but its down-regulation was found in paulownia [17].

The mir172 regulates the Apetala2 gene, and its overexpression could result in the early flowering, development of abnormal flowers, and altered leaf morphology of transgenic plants [53]. In phytoplasma-infected plants, its increased expression was observed but could not be validated with the Northern blot analysis. The reason for the failure could be that only a stem-specific RNA sample was probed, which could show a different expression pattern. It is interesting to note that in *Z. jujube*, a decrease was also noted in the miR171 level, which was found during phytoplasma infection [19].

The miR390 and miR393 regulates auxin-based lateral root development, which can be altered by different types of stresses [54]. It appears that during phytoplasma infection, their levels decrease, but the expression changes. This would be better investigated in the roots to find out the precise mechanism.

It is quite interesting that the miR395 level was highly altered in the phytoplasma-infected plants and that its increase was also reported in an aster yellows-infected grapevine [20]. Furthermore, it plays a role in sulphate assimilation and redox signaling [55]. As its level was markedly changed, its role could be important during the phytoplasma infection, as well as in the development of phytoplasma-specific symptoms.

The miR396 regulates targets that belong to the growth-regulating factor (GRF) family of transcription factors, thereby controlling the cell proliferation in *Arabidopsis* leaves [56]. Its decreased level could be found in the phytoplasma-infected leaves, suggesting its possible role in the development of the symptoms.

The miR398 targets Cu/Zn superoxide dismutase, which is responsible for scavenging reactive oxygen species and for being produced during various biotic stresses, including during bacterial, fungal, and viral infections. Interestingly, the level of the miR398 is usually up-regulated during viral infection; meanwhile, it could be either up- or down-regulated during bacterial infection [57]. Its increased expression was reported in *Z. jujube* [19], but its decrease was shown in the phytoplasma-infected mulberry [15]. Here, the up-regulation of the miR398 and in one of the SOD (CSD2) during phytoplasma infection were found, suggesting that this pathway can also play a role in the defense reaction of the plant.

The expression patterns of novel *C. roseus* miRNA were also changed. This change was remarkable in the case of the cro-novel-34. However, due to the lack of detailed annotations of the genome, it was only possible to speculate whether its induced expression could play a role in the symptom development during phytoplasma infection.

In the phytoplasma-infected plants, the induced level of ORCA2, ORCA3, and CR1 were found, which could result in an increased TIA level. ORCA2, ORCA3, and CR1 can be jasmonate-induced and, as they contain AP2-like motifs in their promoter, it is possible that they are further controlled by the miR172. In parallel, it was found that a decreased level of siRNAs—mapped to the MAT and D4H-like promoter, which could induce the expression of these genes—led to a similarly increased TIA level; however, this point should be further investigated.

Phytoplasma infection highly interferes with the hormone balance and is associated with developmental changes in plants. MiRNAs and siRNAs can play a role during this altered regulation. The pathogen itself is confined to the phloem in an uneven concentration. This spatially different expression of the pathogen can further change temporally during the infection cycle, thus making it particularly difficult to precisely monitor the molecular mechanisms lying behind the symptom expression. An miRNA-based hormone regulation can be one of the main components of this complex process. *C. roseus* is not only the model plant for phytoplasma research, but it is also widely used as a medicinal plant; this is why it is very interesting to know whether the production of its important alkaloids can be affected during phytoplasma infection. The alkaloid content of the phytoplasma-infected plants would be important to further investigate in the future not only to better understand this important process, but also to potentially increase the yield of the beneficial compounds.

## Figures and Tables

**Table 1:** A summary of the main changes in the miRNA expression of the phytoplasma-infected plants, ordered according to the miRNA families. The average RPM values of the most abundant miRNA species are listed together with their relative expression in the Vir and WB plants when compared to the NO plants. Blue shows down-regulation, while red shows up-regulation. Their color intensity is proportional to the intensity of the change.

miRNA	miRNA	Average RPM of the miRNA			Relative Expression of the miRNA		Cluster
		Vir	WB	NO	Vir/NO	WB/NO	
**156**	ath-miR156a-5p	77.1	33.2	46.8	1.65	0.71	5
**157**	ath-miR157a-5p	503.0	569.3	523.7	0.961	1.087	1
	cro-miR157d-3p	7.8	24.6	13.8	0.568	1.785	7
**159**	cro-miR159a	5998.2	3018.2	7460.1	0.804	0.405	4
**162**	cro-miR162a-3p	111.5	121.4	184.3	0.605	0.658	6
**165**	cro-miR165a-3p-2	87.5	118.0	163.3	0.536	0.723	8
**166**	cro-miR166b	589.7	455.5	392.5	1.502	1.160	3
	cro-miR166h-3p	110,187.1	89,050.1	110,680.9	0.996	0.805	4
	cro-miR166	59,129.1	35,752.1	49,446.0	1.196	0.723	5
	cro-miR166e-3p-1	1133.9	1064.7	1105.3	1.026	0.963	5
**167**	ath-miR167d	26.2	26.4	49.7	0.527	0.532	6
	ath-miR167a-5p	15.7	29.3	40.1	0.393	0.730	8
**168**	ath-miR168a-5p	318.1	551.3	1090.5	0.292	0.505	8
**170**	cro-miR170-5p	10.4	12.0	19.4	0.534	0.615	6
**171**	cro-miR171c-5p	8.9	2.2	0.4	21.486	5.307	3
**172**	cro-miR172g-3p	19.1	29.0	6.8	2.823	4.271	2
**319**	cro-miR319e	97.7	69.0	83.3	1.172	0.827	5
	cro-miR319a	51.1	18.2	38.7	1.320	0.470	5
**390**	cro-miR390a-5p	6.3	9.2	20.4	0.310	0.450	6
	cro-miR390a-3p-1	3.9	15.3	27.8	0.142	0.551	8
	cro-miR390a-3p-2	3.8	14.8	27.1	0.142	0.547	8
**391**	gma-miR391-5p	11.4	25.3	27.7	0.412	0.916	8
**395**	cro-miR395a	12.6	1.4	0.3	40.356	4.398	3
	cro-miR395d	12.5	1.3	0.2	57.137	5.850	3
**396**	cro-miR396e	4137.7	1321.1	4099.4	1.009	0.322	4
	cro-miR396b-5p	1287.5	599.6	1283.4	1.003	0.467	4
	cro-miR396h	3291.7	752.5	1779.8	1.849	0.423	5
**398**	cro-miR398	1283.5	976.9	362.3	3.543	2.696	3
**novel**	cro-novel-34	2117.2	2422.3	992.7	2.133	2.440	2

## Data Availability

The FASTQ files of the sequenced libraries were deposited to the NCBI GEO database and can be accessed through the series accession number GSE213754.

## Supplementary Materials

The following supporting information can be downloaded at https://www.mdpi.com/article/10.3390/genes14051114/s1 , Figure S1: Photos about the groups of periwinkle plants used for small RNA NGS, showing different symptoms; Figure S2: A column diagram of the size distribution of the sequenced small RNA reads in the nine sequenced small RNA libraries; Figure S3: A column diagram of the size distribution of the AY-phytoplasma-mapped reads in the sequenced small RNA libraries; Figure S4: Summarized data (a) and Northern blot-based validation of the miR156 (b) and miR157 (c) expressions; Figure S5: Summarized data (a) and Northern blot-based validation of the miR159 (b), miR167 (c), and miR319 (d) expressions; Figure S6: Northern blot-based validation (a) of the expression changes (b) in the miR166 family; Figure S7: Northern blot-based validation (b) of the expression changes (a) in miR168. (c) The revised annotation of the C. roseus miR168 and its precursor; Figure S8: Northern blot-based validation of the expression changes (a) in miR171 (b); Figure S9: Northern blot-based validation of expression changes (a) in miR172 (b); Figure S10: Northern blot-based validation of the expression changes (a) of miR390 (b); Figure S11: Northern blot-based validation (a) of the expression changes (b) in miR396; Figure S12: Northern blot-based validation (a) of the expression changes (b) in miR398; Table S1: Initial statistics of the sequenced small RNA libraries; Table S2: The RPM values of the miRNAs in the different libraries and their average RPM in the three different groups of plants, ordered according to the miRNA families; Table S3: The z-scores of the miRNAs in the three different groups of plants, showing the data for the detected miRNA and ordered according to the clusters shown in Figure 4; Table S4: Summary of the main changes in the miRNA expression of the phytoplasma-infected plants; Table S5: The z-scores of the siRNAs in the three different groups of plants, showing the data for the detected siRNA and ordered according to the clusters shown in Figure 6; Table S6: The sequences of the primers used for the RT-qPCR or Northern blot hybridization.

## References

[1] IRPCM (2004). ‘Candidatus Phytoplasma’, a taxon for the wall-less, non-helical prokaryotes that colonize plant phloem and insects. Int. J. Syst. Evol. Microbiol..

[2] Bertaccini A. (2022). Plants and Phytoplasmas: When Bacteria Modify Plants. Plants.

[3] Dermastia M. (2019). Plant Hormones in Phytoplasma Infected Plants. Front. Plant Sci..

[4] Bendix C., Lewis J.D. (2018). The enemy within: Phloem-limited pathogens. Mol. Plant Pathol..

[5] Chen X. (2005). MicroRNA biogenesis and function in plants. FEBS Lett..

[6] Szittya G., Burgyán J. (2013). RNA interference-mediated intrinsic antiviral immunity in plants. Curr. Top Microbiol. Immunol..

[7] Pumplin N., Voinnet O. (2013). RNA silencing suppression by plant pathogens: Defence, counter-defence and counter-counter-defence. Nat. Rev. Microbiol..

[8] Wang L., Chen W., Ma H., Li J., Hao X., Wu Y. (2021). Identification of RNA silencing suppressor encoded by wheat blue dwarf (WBD) phytoplasma. Plant Biol..

[9] Kaviani M., Goodwin P.H., Hunter D.M. (2022). Differences in Gene Expression of Pear Selections Showing Leaf Curling or Leaf Reddening Symptoms Due to Pear Decline Phytoplasma. Plants.

[10] Tan Y., Li Q., Zhao Y., Wei H., Wang J., Baker C.J., Liu Q., Wei W. (2021). Integration of metabolomics and existing omics data reveals new insights into phytoplasma-induced metabolic reprogramming in host plants. PLoS ONE.

[11] Wang X., Hu Q., Wang J., Lou L., Xu X., Chen X. (2022). Comparative Biochemical and Transcriptomic Analyses Provide New Insights into Phytoplasma Infection Responses in Cucumber. Genes.

[12] Dermastia M., Škrlj B., Strah R., Anžič B., Tomaž Š., Križnik M., Schönhuber C., Riedle-Bauer M., Ramšak Ž., Petek M. (2021). Differential Response of Grapevine to Infection with ‘Candidatus Phytoplasma solani’ in Early and Late Growing Season through Complex Regulation of mRNA and Small RNA Transcriptomes. Int. J. Mol. Sci..

[13] Liu C., Dong X., Xu Y., Dong Q., Wang Y., Gai Y., Ji X. (2021). Transcriptome and DNA Methylome Reveal Insights Into Phytoplasma Infection Responses in Mulberry (*Morus multicaulis* Perr.). Front. Plant Sci..

[14] Cao X., Zhai X., Zhao Z., Deng M., Li Y., Fan G. (2020). Genome-wide DNA methylation analysis of paulownia with phytoplasma infection. Gene.

[15] Ehya F., Monavarfeshani A., Mohseni Fard E., Karimi Farsad L., Khayam Nekouei M., Mardi M., Salekdeh G.H. (2013). Phytoplasma-Responsive microRNAs Modulate Hormonal, Nutritional, and Stress Signalling Pathways in Mexican Lime Trees. PLoS ONE.

[16] Gai Y.P., Li Y.Q., Guo F.Y., Yuan C.Z., Mo Y.Y., Zhang H.L., Wang H., Ji X.L. (2014). Analysis of phytoplasma-responsive sRNAs provide insight into the pathogenic mechanisms of mulberry yellow dwarf disease. Sci. Rep..

[17] Fan G., Cao X., Niu S., Deng M., Zhao Z., Dong Y. (2015). Transcriptome, microRNA, and degradome analyses of the gene expression of Paulownia with phytoplamsa. BMC Genom..

[18] Fan G., Niu S., Xu T., Deng M., Zhao Z., Wang Y., Cao L., Wang Z. (2015). Plant-Pathogen Interaction-Related MicroRNAs and Their Targets Provide Indicators of Phytoplasma Infection in *Paulownia tomentosa* × *Paulownia fortunei*. PLoS ONE.

[19] Shao F., Zhang Q., Liu H., Lu S., Qiu D. (2016). Genome-Wide Identification and Analysis of MicroRNAs Involved in Witches’-Broom Phytoplasma Response in *Ziziphus jujuba*. PLoS ONE.

[20] Snyman M.C., Solofoharivelo M.C., Souza-Richards R., Stephan D., Murray S., Burger J.T. (2017). The use of high-throughput small RNA sequencing reveals differentially expressed microRNAs in response to aster yellows phytoplasma-infection in *Vitis vinifera* cv. ‘Chardonnay’. PLoS ONE.

[21] Chitarra W., Pagliarani C., Abbà S., Boccacci P., Birello G., Rossi M., Palmano S., Marzachì C., Perrone I., Gambino G. (2018). miRVIT: A Novel miRNA Database and Its Application to Uncover Vitis Responses to Flavescence dorée Infection. Front. Plant Sci..

[22] Wang Q., Xing S., Pan Q., Yuan F., Zhao J., Tian Y., Chen Y., Wang G., Tang K. (2012). Development of efficient *Catharanthus roseus* regeneration and transformation system using agrobacterium tumefaciens and hypocotyls as explants. BMC Biotechnol..

[23] Li C.Y., Leopold A.L., Sander G.W., Shanks J.V., Zhao L., Gibson S.I. (2013). The ORCA2 transcription factor plays a key role in regulation of the terpenoid indole alkaloid pathway. BMC Plant Biol..

[24] Laflamme P., St-Pierre B., De Luca V. (2001). Molecular and Biochemical Analysis of a Madagascar Periwinkle Root-Specific Minovincinine-19-Hydroxy-O-Acetyltransferase1. Plant Physiol..

[25] Liu J., Gao F., Ren J., Lu X., Ren G., Wang R. (2017). A Novel AP2/ERF Transcription Factor CR1 Regulates the Accumulation of Vindoline and Serpentine in *Catharanthus roseus*. Front. Plant Sci..

[26] Pani A., Mahapatra R.K. (2013). Computational identification of microRNAs and their targets in *Catharanthus roseus* expressed sequence tags. Genom. Data.

[27] Prakash P., Ghosliya D., Gupta V. (2015). Identification of conserved and novel microRNAs in *Catharanthus roseus* by deep sequencing and computational prediction of their potential targets. Gene.

[28] Shen E.M., Singh S.K., Ghosh J.S., Patra B., Paul P., Yuan L., Pattanaik S. (2017). The miRNAome of *Catharanthus roseus*: Identification, expression analysis, and potential roles of microRNAs in regulation of terpenoid indole alkaloid biosynthesis. Sci. Rep..

[29] Bertaccini A., Davis R.E., Lee I.M. (1992). In Vitro Micropropagation for Maintenance of Mycoplasma-like Organisms in Infected Plant Tissues. HortScience.

[30] Bertaccini A. (2014). Phytoplasma Collection. https://www.ipwgnet.org/collection.

[31] White J.L., Kaper J.M. (1989). A simple method for detection of viral satellite RNAs in small plant tissue samples. J. Virol. Methods.

[32] Czotter N., Molnár J., Pesti R., Demián E., Baráth D., Varga T., Várallyay É. (2018). Use of siRNAs for Diagnosis of Viruses Associated to Woody Plants in Nurseries and Stock Collections. Methods Mol. Biol..

[33] Kellner F., Kim J., Clavijo B.J., Hamilton J.P., Childs K.L., Vaillancourt B., Cepela J., Habermann M., Steuernagel B., Clissold L. (2015). Genome-guided investigation of plant natural product biosynthesis. Plant J..

[34] Bai X., Zhang J., Ewing A., Miller S.A., Jancso Radek A., Shevchenko D.V., Tsukerman K., Walunas T., Lapidus A., Campbell J.W. (2006). Living with genome instability: The adaptation of phytoplasmas to diverse environments of their insect and plant hosts. J. Bacteriol..

[35] She J., Yan H., Yang J., Xu W., Su Z. (2019). croFGD: *Catharanthus roseus* Functional Genomics Database. Front. Genet.

[36] Kolde R. (2012). Package ‘Pheatmap’. https://cran.r-project.org/web/packages/pheatmap.

[37] Metsalu T., Vilo J. (2015). ClustVis: A web tool for visualizing clustering of multivariate data using Principal Component Analysis and heatmap. Nucleic Acids Res..

[38] Várallyay E., Burgyán J., Havelda Z. (2008). MicroRNA detection by northern blotting using locked nucleic acid probes. Nat. Protoc..

[39] Zambon Y., Contaldo N., Laurita R., Várallyay E., Canel A., Gherardi M., Colombo V., Bertaccini A. (2020). Plasma activated water triggers plant defence responses. Sci. Rep..

[40] Dai X., Zhuang Z., Zhao P.X. (2018). psRNATarget: A plant small RNA target analysis server (2017 release). Nucleic Acids Res..

[41] Mi S., Cai T., Hu Y., Chen Y., Hodges E., Ni F., Wu L., Li S., Zhou H., Long C. (2008). Sorting of small RNAs into *Arabidopsis* argonaute complexes is directed by the 5′ terminal nucleotide. Cell.

[42] Zhou C., Zhang J., Zhao S.J., Hu Z.B. (2014). An active Catharanthus roseus desacetoxyvindoline-4-hydroxylase-like gene and its transcriptional regulatory profile. Bot. Stud..

[43] Peebles C.A., Hughes E.H., Shanks J.V., San K.Y. (2009). Transcriptional response of the terpenoid indole alkaloid pathway to the overexpression of ORCA3 along with jasmonic acid elicitation of *Catharanthus roseus* hairy roots over time. Metab. Eng..

[44] Li Y., Alonso-Peral M., Wong G., Wang M.B., Millar A.A. (2016). Ubiquitous miR159 repression of MYB33/65 in *Arabidopsis* rosettes is robust and is not perturbed by a wide range of stresses. BMC Plant Biol..

[45] Zhu C., Ding Y., Liu H. (2011). MiR398 and plant stress responses. Physiol. Plant..

[46] Su Y.T., Chen J.C., Lin C.P. (2011). Phytoplasma-induced floral abnormalities in *Catharanthus roseus* are associated with phytoplasma accumulation and transcript repression of floral organ identity genes. Mol. Plant Microbe Interact..

[47] Gai Y.P., Zhao H.N., Zhao Y.N., Zhu B.S., Yuan S.S., Li S., Guo F.Y., Ji X.L. (2018). MiRNA-seq-based profiles of miRNAs in mulberry phloem sap provide insight into the pathogenic mechanisms of mulberry yellow dwarf disease. Sci. Rep..

[48] Zheng C., Ye M., Sang M., Wu R. (2019). A Regulatory Network for miR156-SPL Module in *Arabidopsis thaliana*. Int. J. Mol. Sci..

[49] Wu M.F., Tian Q., Reed J.W. (2006). *Arabidopsis* microRNA167 controls patterns of ARF6 and ARF8 expression, and regulates both female and male reproduction. Development.

[50] Turchi L., Baima S., Morelli G., Ruberti I. (2015). Interplay of HD-Zip II and III transcription factors in auxin-regulated plant development. J. Exp. Bot..

[51] Li X.P., Ma X.C., Wang H., Zhu Y., Liu X.X., Li T.T., Zheng Y.P., Zhao J.Q., Zhang J.W., Huang Y.Y. (2020). Osa-miR162a fine-tunes rice resistance to Magnaporthe oryzae and Yield. Rice.

[52] Várallyay E., Válóczi A., Agyi A., Burgyán J., Havelda Z. (2010). Plant virus-mediated induction of miR168 is associated with repression of ARGONAUTE1 accumulation. Embo J..

[53] Tang M., Bai X., Niu L.J., Chai X., Chen M.S., Xu Z.F. (2018). miR172 Regulates both Vegetative and Reproductive Development in the Perennial Woody Plant *Jatropha curcas*. Plant Cell Physiol..

[54] Lu Y., Feng Z., Liu X., Bian L., Xie H., Zhang C., Mysore K.S., Liang J. (2018). MiR393 and miR390 synergistically regulate lateral root growth in rice under different conditions. BMC Plant Biol..

[55] Liang G., Yang F., Yu D. (2010). MicroRNA395 mediates regulation of sulfate accumulation and allocation in *Arabidopsis thaliana*. Plant J..

[56] Debernardi J.M., Rodriguez R.E., Mecchia M.A., Palatnik J.F. (2012). Functional specialization of the plant miR396 regulatory network through distinct microRNA-target interactions. PLoS Genet..

[57] Li J., Song Q., Zuo Z.F., Liu L. (2022). MicroRNA398: A Master Regulator of Plant Development and Stress Responses. Int. J. Mol. Sci..

