# Supplementary material for: Small RNA Profiling of Aster Yellows Phytoplasma-Infected Catharanthus roseus Plants Showing Different Symptoms
Source: Genes (Basel). 2023 May 19;14(5):1114. doi: 10.3390/genes14051114 (PMC10218541; doi:10.3390/genes14051114)
Supplement: Supplementary file 1 [file genes-14-01114-s001.zip › Contaldo et al_Genes_SFigures.pdf]

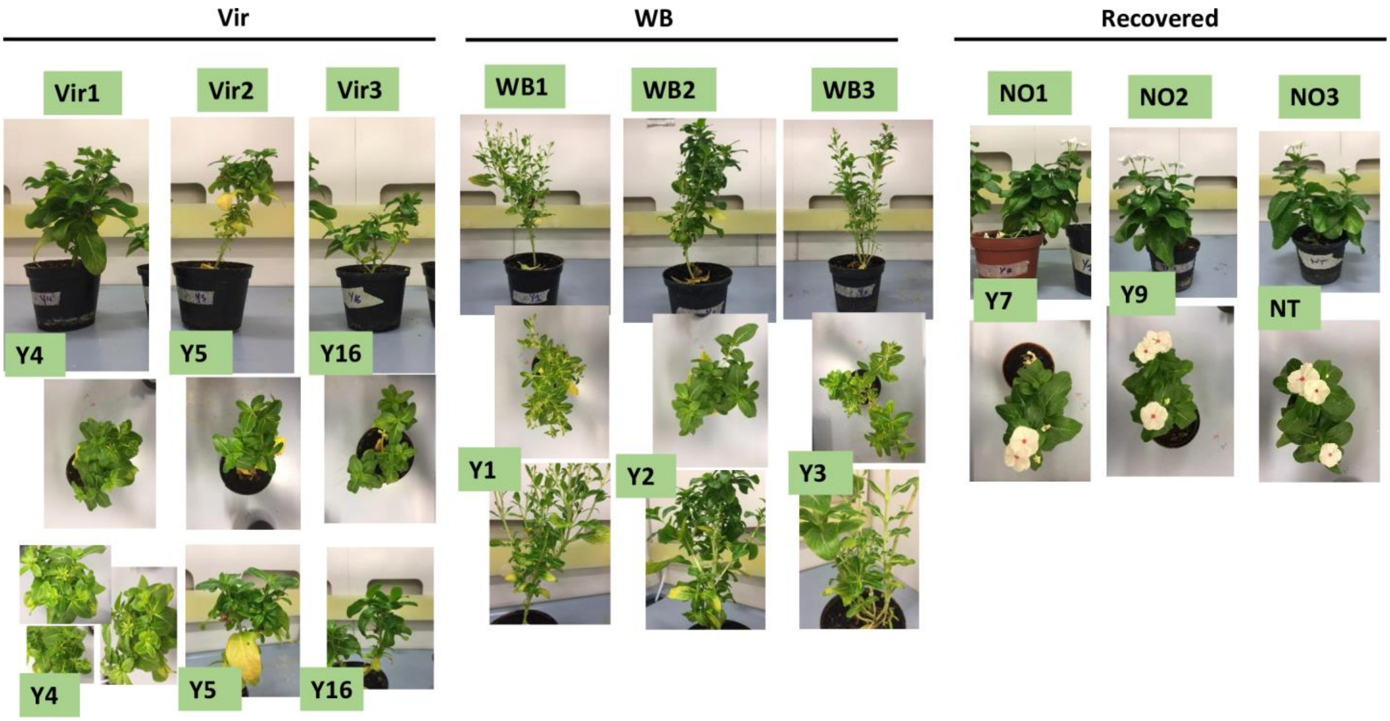

Figure S1: Photos about the groups of periwinkle plants used for small RNA HTS, showing different symptoms: virescence (Vir), witches' broom (WB) and asymptomatic (NO).

(a)

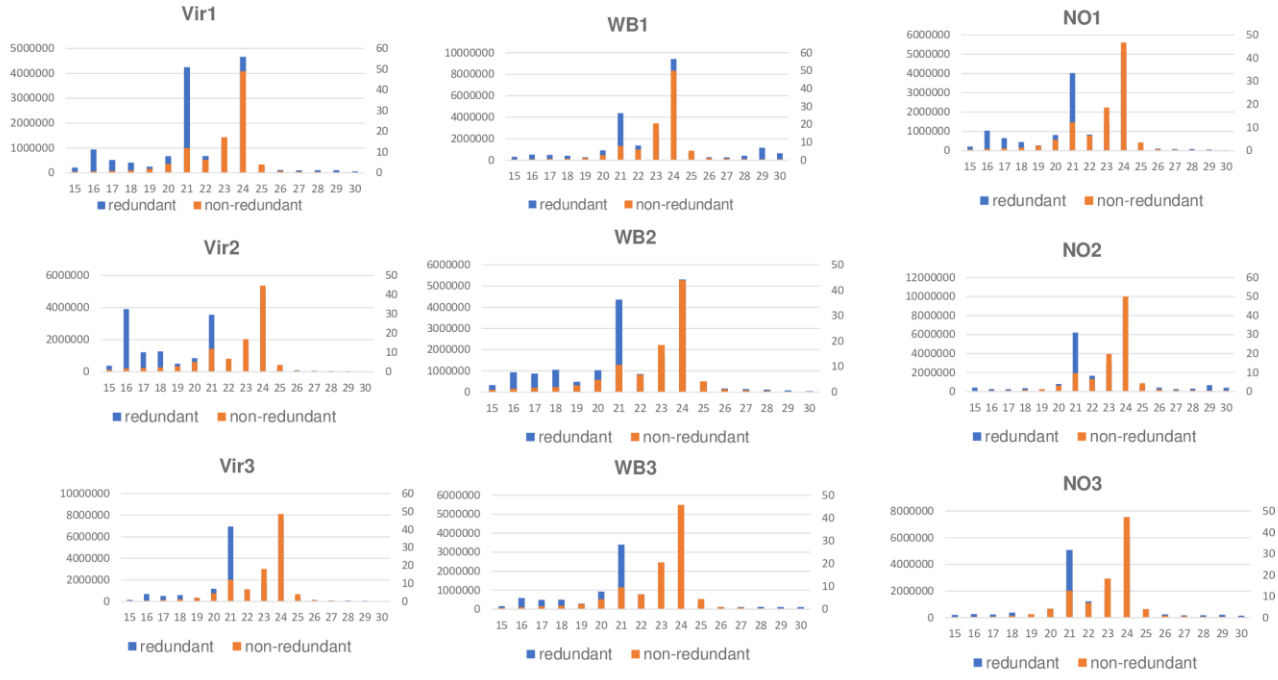

(b)

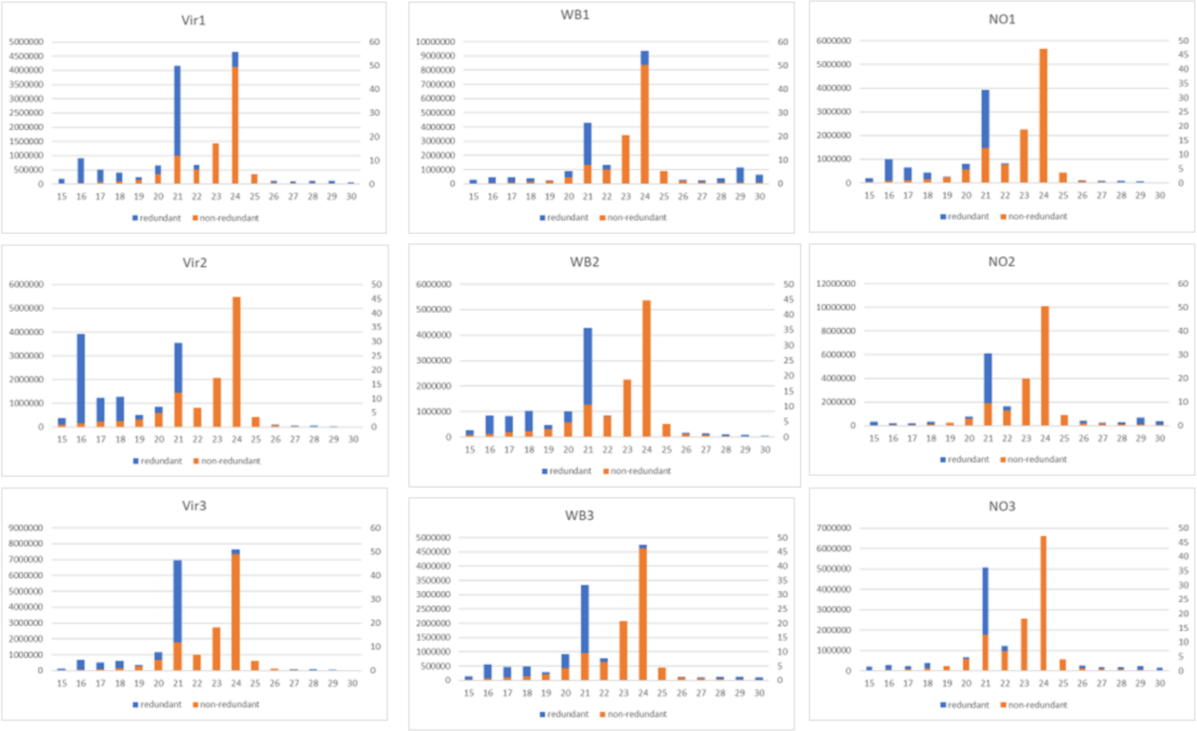

Figure S2. A column diagram of the size distribution of the sequenced and *C.roseus* genome mapped small RNA reads in the nine sequenced small RNA libraries (a) Column diagram of the size distribution of the sequenced small RNA reads in the nine sequenced small RNA libraries, showing number of redundant reads (blue) and percentage of the indicated size-class of non-redundant reads (orange). (b) Column diagram of the size distribution of the *C. roseus* mapped sequenced small RNA reads in the nine sequenced small RNA libraries, showing number of redundant reads (blue) and percentage of the indicated size-class of non-redundant reads (orange).

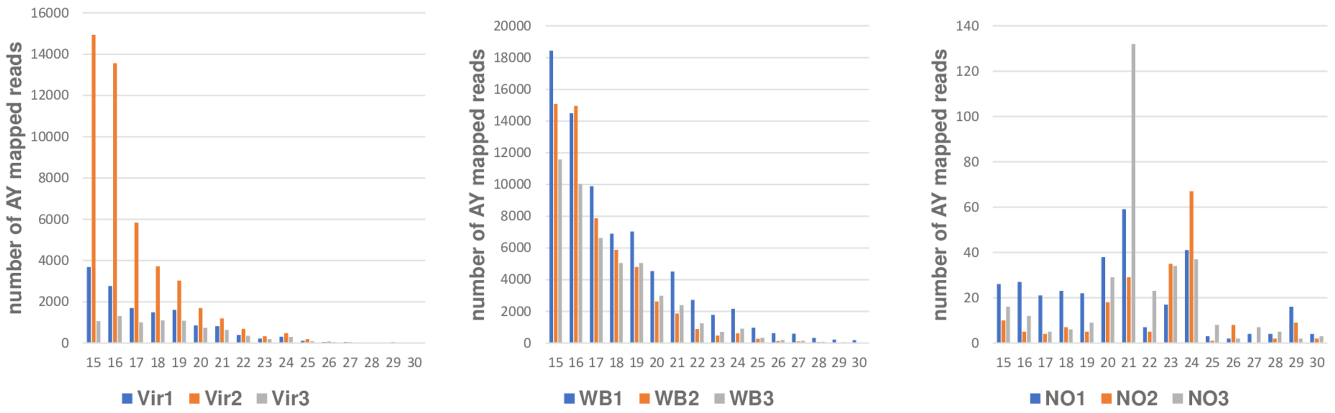

Figure S3. A column diagram of the size distribution of the AY-phytoplasma-mapped reads in the sequenced small RNA libraries .

(a)

	miRNA	Vir	WB	NO	Vir/NO	WB/NO	Clusters
156	ath-miR156a-5p	77,1125683	33,2043382	46,78088036	1,648377878	0,70978438	5
	cro-miR156b-3p-2	5,92791852	5,29208693	9,550221035	0,620710086	0,554132403	6
	cro-miR156f-3p-1	6,077344537	5,23255333	9,449257104	0,643155803	0,553752879	6
	cro-miR156d-3p	7,917046155	24,6650301	13,84918101	0,571661685	1,780973914	7
157	ath-miR157a-5p	503,0351438	569,296051	523,7016851	0,96053757	1,087061713	1
	cro-miR157d-3p	7,838819259	24,6425626	13,80481865	0,567832107	1,785069635	7

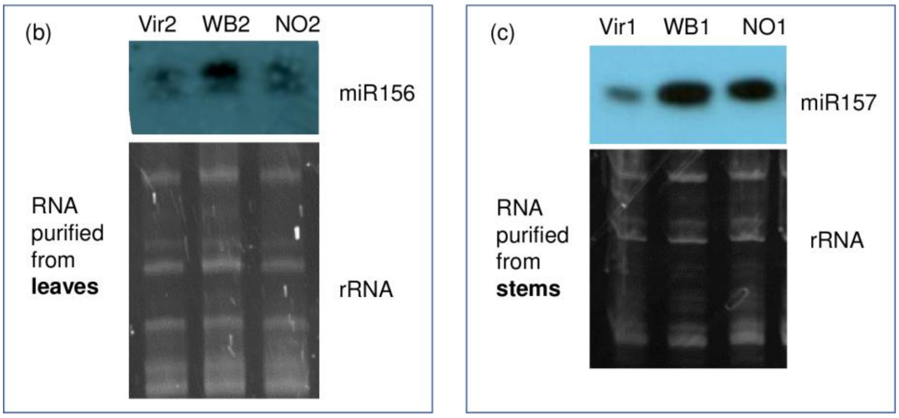

Figure S4. Summarized data (a) and Northern-blot-based validation of the miR156 (b) and miR157 (c) expressions (a) RPM of miRNA sequenced in the different libraries (indicated as the average of results in three different libraries) and relative expression (RMP in the Vir or WB libraries compared to the RPM of the same miRNA in NO libraries) green indicates, the most abundant form of the miRNA family. Down-regulation in the symptomatic plants is indicated with blue, while up-regulation is indicated with red colour. The intensity of the colour is proportional to the intensity of the change. (b) Northern blot hybridization using radioactively labelled LNA probe detecting the indicated miRNA in the extract of one of the Vir, WB and NO plants. EtBr-stained gel serves as a loading control.

(a)

	miRNA	Vir	WB	NO	Vir/NO	WB/NO	Clusters
159	cro-miR159a	5998,228	3018,225	7460,135	0,804037	0,40458	4
	cro-miR159b	195,4496	127,9513	240,9146	0,811282	0,531107	4
	cro-miR159f	27,205	16,45768	38,71216	0,702751	0,42513	4
	ath-miR159b-3p	6,49863	4,417747	6,245412	1,040545	0,707359	4
	gma-miR159d	4,422338	3,01732	5,160942	0,856886	0,584645	4
	ath-miR159c	22,36534	14,7307	20,2906	1,102251	0,725986	5
	gma-miR159a-5p	10,54293	20,8718	22,10483	0,476952	0,944219	8
167	ath-miR167d	26,21693	26,44958	49,72351	0,527254	0,531933	6
	ath-miR167a-5p	15,72634	29,25004	40,05122	0,392656	0,730316	8
	gma-miR167l	3,021233	5,032893	7,117735	0,424465	0,707092	8
319	cro-miR319e	97,68813	68,95573	83,3405	1,172157	0,827398	5
	cro-miR319a	51,13002	18,19865	38,72635	1,32029	0,46993	5
	cro-miR319a-3p	23,60829	11,32947	14,22525	1,659605	0,796434	5
	cro-miR319g	23,39652	11,13625	14,01016	1,669968	0,79487	5
	cro-miR319	5,200613	4,409246	7,443029	0,698723	0,592399	6

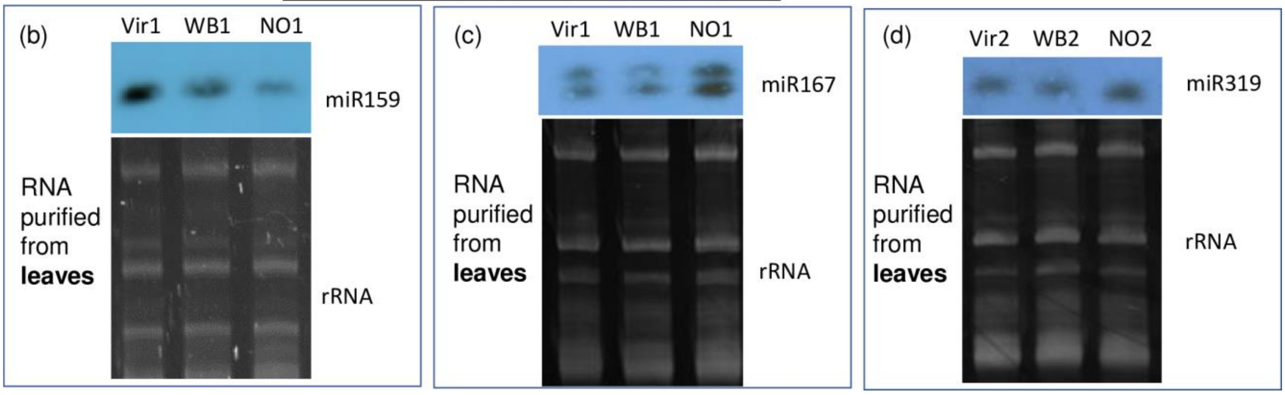

Figure S5. Summarized data (a) and Northern-blot-based validation of the miR159 (b), miR167 (c), and miR319 (d) expressions (a) RPM of miRNA sequenced in the different libraries (indicated as the average of results in three libraries) and relative expression (RMP in the Vir or WB libraries compared to the RPM of the same miRNA in NO libraries) green indicates, the most abundant form of the miRNA family. Down-regulation in the symptomatic plants is indicated with blue, while up-regulation is indicated with red colour. (b) Northern blot hybridization using radioactively labelled LNA probe detecting the indicated miRNA in the extract of one of the Vir, WB and NO plants. EtBr-stained gel serves as a loading control.

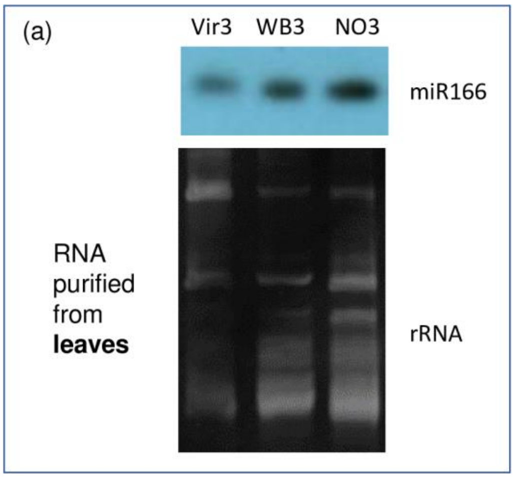

(b)

	miRNA	Vir	WB	NO	Vir/NO	WB/NO	Clusters
166	cro-miR166h-5p	17,54221	21,18912	16,5634	1,059095	1,279274	1
	cro-miR166d-5p	9,516251	23,1809	11,96897	0,795077	1,93675	1
	gma-miR166i-5p	7,549301	25,71514	3,571171	2,113957	7,200758	1
	ath-miR166e-5p	4,349219	5,077847	3,273827	1,328482	1,551043	2
	cro-miR166b-5p	3,402947	5,238144	1,840956	1,848468	2,845339	2
	cro-miR166b	589,6674	455,4685	392,4807	1,502411	1,160486	3
	cro-miR166m	581,5013	450,1241	386,5643	1,504281	1,164422	3
	cro-miR166a	579,3196	449,0037	383,8555	1,509213	1,169721	3
	cro-miR166j-3p	576,7013	447,3575	381,7568	1,510651	1,171839	3
	cro-miR166T	214,1349	181,5423	171,6548	1,247474	1,057601	3
	gma-miR166h-3p	40,78944	32,37299	30,07856	1,356097	1,076281	3
	cro-miR166h-3p	110187,1	89050,09	110680,9	0,995538	0,804566	4
	cro-miR166e-3p-2	21,12882	14,77526	21,17174	0,997973	0,697877	4
	cro-miR166	59129,09	35752,06	49445,98	1,195832	0,723053	5
	cro-miR166e-3p-1	1133,944	1064,745	1105,346	1,025873	0,963269	5
	cro-miR166i	62,04765	38,94707	51,43605	1,206307	0,757194	5
	sly-miR166c-3p	14,35843	7,908576	11,24505	1,276867	0,703294	5
	gma-miR166p	8,477161	6,821987	7,407323	1,14443	0,920979	5
	cro-miR166c	47,23598	43,21814	65,52246	0,720913	0,659593	6
	ath-miR166a-3p	8,405639	13,43659	24,91604	0,337359	0,539275	8

Figure S6. Northern-blot-based validation (a) of the expression changes (b) in the miR166 family (a) Northern blot hybridization using radioactively labelled LNA probe detecting the indicated miRNA in the extract of one of the Vir, WB and NO plants. EtBr-stained gel serves as a loading control. (b) RPM of miRNA sequenced in the different libraries (indicated as the average of results in three libraries) and relative expression (RMP in the Vir or WB libraries compared to the RPM of the same miRNA in NO libraries) green indicates, the most abundant form of the miRNA family. MiRNA forms indicated with grey have identical sequences. Down-regulation in the symptomatic plants is indicated with blue, while up-regulation is indicated with red colour.

(a)

	miRNA	Vir	WB	NO	Vir/NO	WB/NO	Clusters
168	ath-miR168a-5p	318,1326	551,2538	1090,538	0,291721	0,505488	8
	cro-miR168a-3p-1	9,618233	16,36525	32,7588	0,293608	0,499568	8
	gma-miR168b	11,91938	23,77552	26,21469	0,454683	0,906954	8

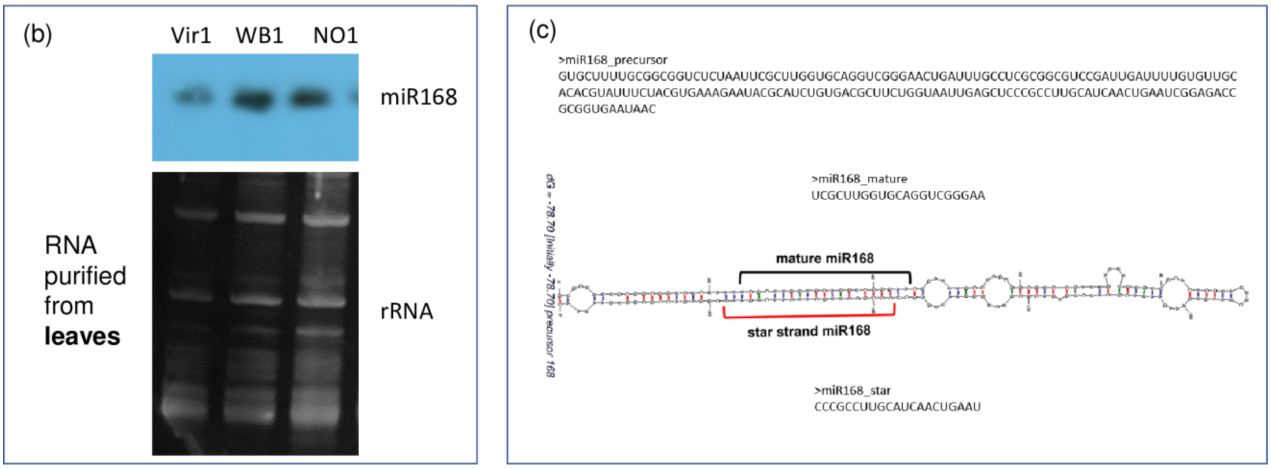

Figure S7. Northern-blot-based validation (b) of the expression changes (a) in miR168. (c) The revised annotation of the C. roseus miR168 and its precursor (a) RPM of miRNA sequenced in the different libraries (indicated as the average of results in three libraries) and relative expression (RMP in the Vir or WB libraries compared to the RPM of the same miRNA in NO libraries) green indicates, the most abundant form of the miRNA family. Down-regulation in the symptomatic plants is indicated with blue, while up-regulation is indicated with red colour. (b) Northern blot hybridization using radioactively labelled LNA probe detecting the indicated miRNA in the extract of one of the Vir, WB and NO plants. EtBr-stained gel serves as a loading control. (c) Proper sequence of the miR168 precursor and the mature miR168 miRNA, together with the schematic draw of the stem-loop structure of the miR168 precursor.

(a)

	miRNA	Vir	WB	NO	Vir/NO	WB/NO	Clusters
171	cro-miR171c-5p	8,870263	2,191071	0,412837	21,48613	5,307353	3

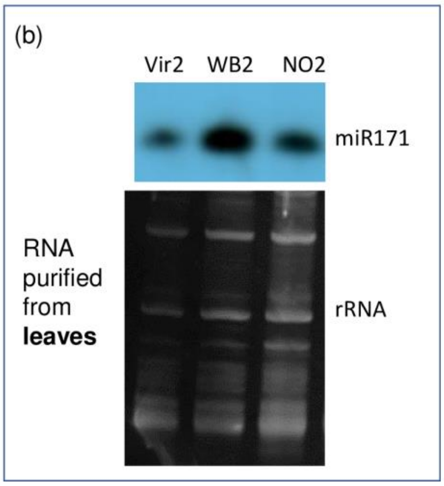

Figure S8. Northern-blot-based validation of the expression changes (a) in miR171 (b). (a) RPM of miRNA sequenced in the different libraries (indicated as the average of results in three libraries) and relative expression (RMP in the Vir or WB libraries compared to the RPM of the same miRNA in NO libraries) green indicates, the most abundant form of the miRNA family. Down-regulation in the symptomatic plants is indicated with blue, while up-regulation is indicated with red colour. (b) Northern blot hybridization using radioactively labelled LNA probe detecting the indicated miRNA in the extract of one of the Vir, WB and NO plants. EtBr-stained gel serves as a loading control.

(a)

	miRNA	Vir	WB	NO	Vir/NO	WB/NO	Clusters
172	cro-miR172g-3p	19,14587	28,96857	6,783105	2,822582	4,270694	2

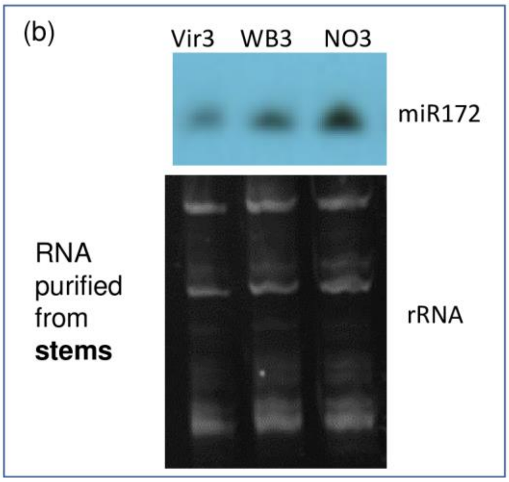

Figure S9. Northern-blot-based validation of expression changes (a) in miR172 (b). (a) RPM of miRNA sequenced in the different libraries (indicated as the average of results in three libraries) and relative expression (RMP in the Vir or WB libraries compared to the RPM of the same miRNA in NO libraries) green indicates, the most abundant form of the miRNA family. Down-regulation in the symptomatic plants is indicated with blue, while up-regulation is indicated with red colour. (b) Northern blot hybridization using radioactively labelled LNA probe detecting the indicated miRNA in the extract of one of the Vir, WB and NO plants. EtBr-stained gel serves as a loading control.

(a)

	miRNA	Vir	WB	NO	Vir/NO	WB/NO	Clusters
390	cro-miR390a-5p	6,310091	9,173722	20,37662	0,309673	0,450208	6
	cro-miR390a-3p-1	3,948597	15,33463	27,82598	0,141903	0,55109	8
	cro-miR390a-3p-2	3,832457	14,78795	27,0569	0,141644	0,54655	8

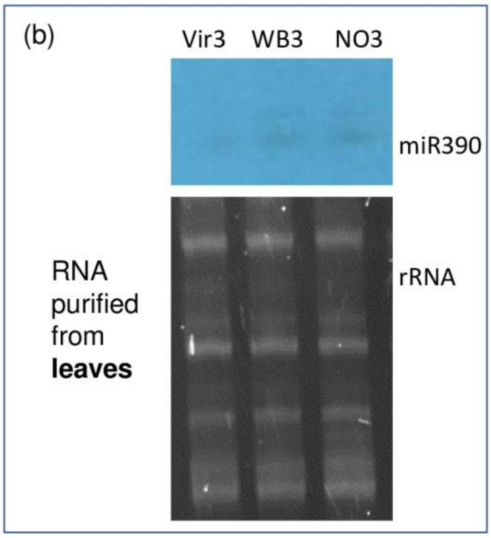

Figure S10. Northern-blot-based validation of the expression changes (a) of miR390 (b). (a) RPM of miRNA sequenced in the different libraries (indicated as the average of results in three libraries) and relative expression (RMP in the Vir or WB libraries compared to the RPM of the same miRNA in NO libraries) green indicates, the most abundant form of the miRNA family. Down-regulation in the symptomatic plants is indicated with blue, while up-regulation is indicated with red colour. (b) Northern blot hybridization using radioactively labelled LNA probe detecting the indicated miRNA in the extract of one of the Vir, WB and NO plants. EtBr-stained gel serves as a loading control.

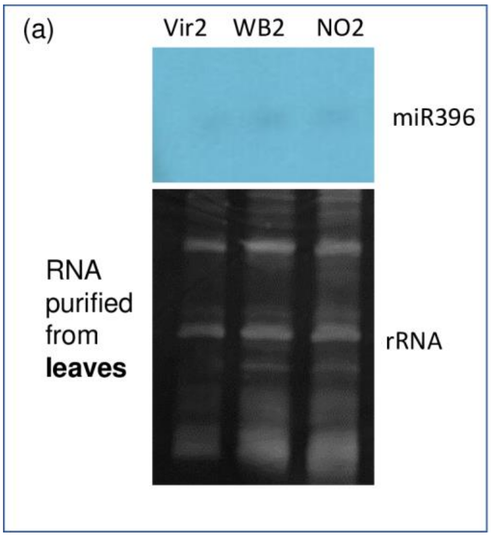

(b)

	miRNA	Vir	WB	NO	Vir/NO	WB/NO	Clusters
396	cro-miR396c	14,82585	17,81277	14,20835	1,04346	1,253683	1
	cro-miR396e	4137,701	1321,144	4099,388	1,009346	0,322278	4
	cro-miR396a-5p	4131,531	1317,805	4091,02	1,009903	0,322121	4
	cro-miR396b-5p	1287,538	599,6436	1283,373	1,003245	0,46724	4
	cro-miR396a-3p-1	102,6773	88,73844	108,391	0,947286	0,818688	4
	cro-miR396g	16,54819	8,838738	22,57042	0,733181	0,391607	4
	cro-miR396b	13,92953	7,878614	19,67106	0,708123	0,400518	4
	cro-miR396a	7,429322	3,467255	9,262614	0,802076	0,374328	4
	cro-miR396d	5,120461	2,784031	7,380429	0,693789	0,377218	4
	cro-miR396h	3291,658	752,5004	1779,812	1,849441	0,422798	5
	cro-miR396a-3p-3	57,69812	37,08528	47,65644	1,21071	0,77818	5

Figure S11. Northern-blot-based validation (a) of the expression changes (b) in miR396. (a) Northern blot hybridization using radioactively labelled LNA probe detecting the indicated miRNA in the extract of one of the Vir, WB and NO plants. EtBr-stained gel serves as a loading control. (b) RPM of miRNA sequenced in the different libraries (indicated as the average of results in three libraries) and relative expression (RMP in the Vir or WB libraries compared to the RPM of the same miRNA in NO libraries) green indicates, the most abundant form of the miRNA family. MiRNA forms indicated with grey have identical sequences. Down-regulation in the symptomatic plants is indicated with blue, while up-regulation is indicated with red colour.

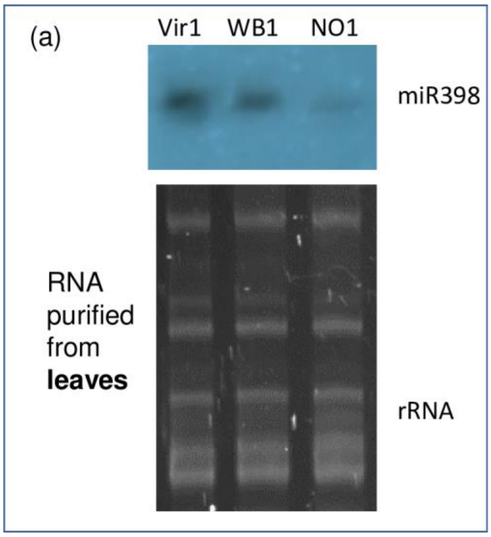

(b)

	miRNA	Vir	WB	NO	Vir/NO	WB/NO	Clusters
398	cro-miR398a-3p-2	1326,381	1020,867	379,618	3,493989	2,689195	3
	cro-miR398f	1301,832	995,0481	368,6059	3,531773	2,699491	3
	cro-miR398a-3p-1	1287,875	985,0677	364,3471	3,534747	2,703652	3
	cro-miR398	1283,456	976,9215	362,2986	3,542535	2,696454	3

Figure S12. Northern-blot-based validation (a) of the expression changes (b) in miR398. (a) Northern blot hybridization using radioactively labelled LNA probe detecting the indicated miRNA in the extract of one of the Vir, WB and NO plants. EtBr-stained gel serves as a loading control. (b) RPM of miRNA sequenced in the different libraries (indicated as the average of results in three libraries) and relative expression (RMP in the Vir or WB libraries compared to the RPM of the same miRNA in NO libraries) green indicates, the most abundant form of the miRNA family. MiRNA forms indicated with grey have identical sequences. Down-regulation in the symptomatic plants is indicated with blue, while up-regulation is indicated with red colour.
